# A humanized monoclonal antibody targeting protein a promotes opsonophagocytosis of Staphylococcus aureus in human umbilical cord blood

**DOI:** 10.1016/j.vaccine.2023.07.018

**Published:** 2023-08-07

**Authors:** Paola Nol Bernardino, Mohini Bhattacharya, Xinhai Chen, Julia Jenkins, Dominique Missiakas, Vilasack Thammavongsa

**Affiliations:** a The University of Chicago, Department of Microbiology, Howard Taylor Ricketts Laboratory, Lemont, IL 60439, USA; b IMMUNARTES LLC, 400 N Aberdeen St, STE 900, Chicago, IL 60642, USA

**Keywords:** *Staphylococcus aureus*, Staphylococcal protein A, Monoclonal antibody, Neonatal bacteremia/meningitis, Antibody-mediated opsonophagocytosis, Umbilical cord blood

## Abstract

Low and very-low-birth-weight (V/LBW) neonates are highly susceptible to bacterial sepsis and meningitis. Bacterial infections caused by Staphylococcus aureus can be particularly dangerous for neonates and can result in high mortality and long-term disabilities. Antibody-based strategies have been attempted to protect V/LBW neonates against staphylococcal disease. However, these efforts have so far been unsuccessful. Failures were attributed to the immaturity of the neonatal immune system but did not account for the anti-opsonic activity of Staphylococcal protein A (SpA). Here we show that monoclonal antibody 3F6, which blocks SpA activity, promotes complement-dependent cell-mediated phagocytosis of *S. aureus* in human umbilical cord blood. A substitution in the crystallizable fragment (Fc) region of 3F6 that enhances recruitment of complement component C1q further increases the phagocytic activity of cord blood. Our data demonstrate that the neonatal immune system possesses bactericidal activity that can be harnessed by antibodies that circumvent a key innate immune strategy of *S. aureus*.

## 1 Introduction

Neonatal infections are a leading cause of morbidity and mortality worldwide, particularly in low and very-low-birth-weight (V/LBW) and preterm babies [1]. These newborns are at increased risk for infections due to their immature immune systems, as well as their exposure to a variety of microorganisms rapidly after birth. Staphylococcus aureus is the second most common cause of late-onset sepsis and meningitis in V/LBW babies admitted to neonatal intensive care units [2] and even with appropriate antibiotic therapy, neonatal infections can lead to long-term neurological and developmental problems [3]. The development of effective strategies to prevent *S. aureus* infections in newborns remain a challenge, as the bacterium is highly adaptive and can develop resistance to antibiotics.

An FDA approved antibody that prevents the incidence of *S. aureus* sepsis/meningitis in newborns and infants is not available. Early studies by C.J. Baker and colleagues suggested that administration of pooled human immunoglobulin (hIg) to V/LBW neonates may have a protective effect against bacterial infection [4]. This intervention however, did not reduce disease incidence or mortality [5]. Further efforts examined the clinical efficacy of human antibodies against staphylococcal capsule (Altastaph®), clumping factor A (ClfA) and serine-aspartate repeat protein G (SdrG) (INH-A21) or monoclonal antibody against polyglycerol-phosphate lipoteichoic acid (Pagibaximab®) [6]. None of these clinical trials met their study endpoints toward the protection of V/LBW neonates against S. aureus infections [6].

We reported earlier that the failure of antibodies to provide protection against *S. aureus* is based on the immune evasive attributes of staphylococcal protein A (SpA). SpA captures the Fcγ domain of IgG molecules as well as Fab domains of V_H_3-type IgG, IgM and IgE molecules [7]. Fcγ binding effectively neutralizes opsonophagocytic IgG antibodies directed against the pathogen. Fab binding and crosslinking of V_H_3-type IgM B cell receptors (BCR) triggers non-specific B cell expansion while crosslinking of IgG/IgE on surface of mast cells and basophils triggers histamine release [7]. Earlier we isolated 3F6-hIgG1, a humanized IgG1 antibody that binds SpA in an immune-dependent manner, promotes killing of *S. aureus* in adult murine and human blood, and protects neonatal and adult mice from *S. aureus* disease [8], [9], [10], [11], [12]. The binding of 3F6-hIgG1 to the surface of *S. aureus* was shown to enhance interactions with Fcγ receptors (FcγR) on innate immune cells and with the six-headed globular domains of complement molecule C1q, promote antibody-dependent cell-mediated phagocytosis (ADCP) and complement-dependent cell-mediated phagocytosis (CDCP), respectively [11], [12].

Passive transfer of SpA-neutralizing antibodies could be a useful addition to conventional antibiotic treatments in newborns at risk of *S. aureus* infection. Human umbilical cord blood cells have been used to compare the capacity of neonatal and adult immune cells to produce immune responses [13]. Here, we use human cord blood as a model to evaluate antibodies that target SpA for their ability to promote phagocytic uptake of *S. aureus* in newborns.

## 2 Materials and methods

### 2.1 Ethics statement

Adult and umbilical cord blood samples were collected from informed, consenting volunteers under approved University of Chicago Institutional Review Board (IRB) protocols. *S. aureus* experiments were conducted under the University of Chicago Institutional Biosafety Committee (IBC) supervision.

### 2.2 Collection of blood

Healthy adult blood was collected in sodium heparin-coated tubes (BDScience), used within 30 min at room temperature. Cord blood, collected post-delivery, was stored in solubilized sodium heparin (30 USP/mL; Sigma-Aldrich) on ice and removed 30 min before bacterial incubation.

### 2.3 Complete blood count (CBC) analysis of blood

Blood was immediately transported to the University of Chicago Hematology Lab for processing. Clotted samples were excluded from analysis. Complete blood counts were obtained using a Sysmex XN-10 analyzer (XN-9100 system).

### 2.4 Bacterial strain

*S. aureus* USA300 LAC, a methicillin-resistant clinical isolate (MRSA), was grown in tryptic soy broth or agar (TSB/TSA) at 37 °C. Overnight cultures were diluted (1:100) into fresh TSB and grown for 3 h at 37 °C. Staphylococci were centrifuged, washed twice and diluted in PBS to A_600_ 0.4 (2 × 10^8^ CFU ml^−1^).

### 2.5 Phagocytic activity in blood

Blood was pre-incubated with cytochalasin D (CD, 0.04 mM) and antibodies (up to 10 μg ml^−1^) for 10 min where indicated. Isotype hIgG1 was from Jackson ImmunoResearch, and 3F6-hIgG1 and 3F6-hIgG1^AESP^ were produced as described [11], [12]. Staphylococcal survival was assessed by mixing a 50 μl *S. aureus* suspension (5 × 10^6^ CFU / PBS) in 0.45 ml anticoagulated blood, incubating at 37 °C for 0 and 60 min, and adding 0.5 ml SK lysis buffer (phosphate-buffered saline [PBS] containing 0.5 % saponin, 100 U streptokinase [SK], 50 μg trypsin, 1 μg DNase, and 5 μg RNase) for 10 min at 37 °C before plating on agar for CFU enumeration.

### 2.6 Staphylococcal antigen matrix (SAM)

Staphylococcal antigens (2 μg each of the following proteins: SpA_KKAA_, a variant unable to bind to Fcγ- and Fab-domains of Ig [14]; ClfA and ClfB; iron-regulated surface determinant A, IsdA; iron-regulated surface determinant B, IsdB; coagulase, Coa; von-Willebrand factor binding protein, vWbp; alpha-hemolysin, Hla; fibronectin binding protein A, FnbpA and FnbpB) were blotted onto nitrocellulose, blocked with 5 % milk and incubated with human plasma. Signal intensities (A_700_) were quantified using IRDye 680-conjugated anti-human IgG (Rockland) and the Odyssey™ infrared imaging system (Li-cor) as described [14].

### 2.7 Blood smear, Giemsa stain and microscopy

Blood mixed with bacteria (1 µl) was smeared on a glass slide, fixed with methanol, and stained with Wright-Giemsa. Stained slides were visualized using a Zeiss Axioscope upright histology microscope.

## 3 Results

### 3.1 Comparing the phagocytic activity of adult and cord blood toward *S. aureus.*

**Fig. 1:**
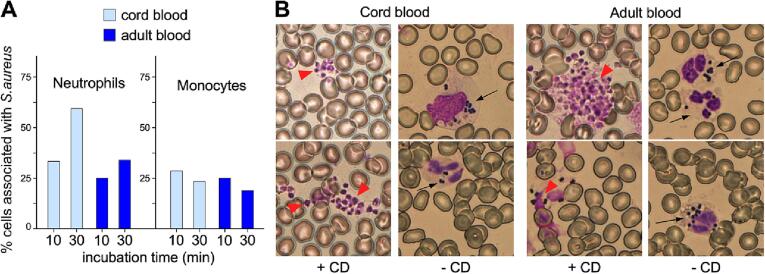
**Phagocytosis of *S. aureus* by immune cells in cord and adult blood.** (A) Association of staphylococci with neutrophils and monocytes in cord or adult blood after incubation for 10 and 30 min. Ten images were collected per time point and approximately 300 immune cells were counted to establish the distribution of cells associated with bacteria. (B) Giemsa stains of whole blood samples incubated with bacteria for 30 min in presence (+CD) and absence (−CD) of cytochalasin D. Red arrows point to extracellular agglutinated staphylococci. Black arrows point to neutrophils filled with bacteria. Data displayed is from 1 of 2 experiments, conducted independently.

Umbilical cord blood provides a window into the *in-utero* environment, containing all components of the innate immune system newborns utilize to fend off infections. We incubated anticoagulated cord blood from full-term newborn deliveries, or peripheral blood from healthy adult donors, with *S. aureus* strain USA300, and then analyzed the association of immune cells with the pathogen. The cellular composition of the cord blood samples did not significantly deviate from reported values (Table 1). *S. aureus* strain USA300 represents the multi-locus sequencing (MLST) sequence type (ST) 8-MRSA-IV, first identified in the United States, but now globally prevalent [15]. After incubation with *S. aureus*, blood smears were stained with Giemsa and visualized by microscopy. Leukocytes were identified based on cellular morphology, and the pattern of immune cells associated with staphylococci was determined. These observations revealed that staphylococci did not associate with lymphocytes or basophils. In comparison to adult blood, cord blood exhibited an increase in the number of staphylococci-associated neutrophils within 10 to 30 min of incubation (Fig. 1A). The association of bacteria with monocytes was similar in both cord blood and adult blood, with no apparent increase after 10- and 30-minute incubation periods (Fig. 1A).

**Table 1:** Differential cell analysis and complement levels of blood specimens.

Cell Type^1^^,^#	Specimen ID^1^									
	M10 (A)	P5 (A)	P4 (A)	M9 (C)	M11 (C)	M3 (C)	P6 (C)	P1 (C)	P2 (C)	P3 (C)
**Neutrophils** (A) 40–70 %, (C) 41–81 %	54.2 %	61.0 %	–	–	–	49.0 %	52.0 %	–	–	–
**Lymphocytes** (A) 20–40 %, (C) 19–36 %	37.4 %	31.0 %	–	–	–	37.0 %	35.0 %	–	–	–
**Monocytes** (A) 2–8 %, (C) 0.4–7 %	5.2 %	6.0 %	–	–	–	7.0 %	5.0 %	–	–	–
**Eosinophils** (A) 1–4 %, (C) 0–2 %	2.2 %	1.0 %	–	–	–	2.0 %	1.5 %	–	–	–
**Basophils** (A) 0.5–1 %, (C) 0–1 %	1.0 %	1.0 %	–	–	–	0.0 %	0.5 %	–	–	–
**Metamyelocyte**	–	–	–	–	–	2.0 %	2.0 %	–	–	–
**Myelocyte**	–	–	–	–	–	3.0 %	4.0 %			
**Absolute Neutrophils** (range in cells × 10^3^/μL) (A) 1.56–6.45, (C) 3–28	2.40	4.53	–	–	–	8.56	7.21	–	–	–
**Absolute Lymphocytes** (range in cells × 10^3^/μL) (A) 0.95–3.07, (C) 2–11	1.70	2.26	–	–	–	6.25	5.13	–	–	–
**Absolute Monocytes** (range in cells × 10^3^/μL) (A) 0.1–1.3, (C) 0.1–4.4	0.20	0.43	–	–	–	1.20	1.30	–	–	–
**Absolute Eosinophils** (range in cells × 10^3^/μL) (A) 0.03–0.3, (C) 0.01–1.5	0.10	0.05	–	–		0.34	0.20	–	–	–
**Absolute Basophils** (range in cells × 10^3^/μL) (A) 0.0–0.2, (C) 0.0–0.7	0.00	0.04	–	–	–	0.00	0.00	–	–	–
**Absolute Metamyelocyte**	–	–	–	–	–	0.26	0.28	–	–	–
**Absolute Myelocyte**	–	–	–	–	–	0.51	0.48	–	–	–
**WBC** (range in cells × 10^3^/μL) (A) 3.5–11, (C) 9.0–35.0	5.9	7.3	–	9.0	11.1	17.1	10.5	–	–	–
**Hemoglobin** (A) 11.5–15.5 g/dL, (C) 14.0–18.8 g/dL	13.0	13.6	–	14.6	11.6	14.7	13.6	–	–	–
**MCV** (A) 81–99 fL, (C) 95–125	91.3	90.9	–	93.0	98.0	111.6	103.2	–	–	–
**RBCDIST WIDTH** (A) < 15 %, (C) 13.0–18.0 %	13.0	12.9	–	13.0	16.9	15.9	12.9	–	–	–
**Platelets count** (A) and (C) 150–450 x10^3^/μL	232.0	254.0	–	170.0	177.0	179.0	189.0	–	–	–

**Complement**										
**C3 (mg/dL)**	–	99	248	–	–	–	97	70	99	94
**C4 (mg/dL)**	–	17	19	–	–	–	50	–	15	13

1 Denotes (A) = adult blood, (C) = cord blood.

# Normal reported values indicated for adult (A) and (C) cord blood.

Most staphylococci were associated with neutrophils and monocytes following the 30-minute incubation; isolated bacteria diffusing freely were seldom observed. We surmised that the majority of bacteria adhere to the cell surface of immune cells or are internalized. The inhibition of phagocytosis by Cytochalasin D (CD), which blocks actin polymerization [16], resulted in the formation of large agglutinated masses of extracellular *S. aureus* (Fig. 1B). This result suggests that phagocytosis must be the primary mechanism underlying the observed association between staphylococci and immune cells.

### 3.2 Phagocytosis of *S. aureus* in cord blood is enhanced with 3F6-hIgG1.

We examined the replication of *S. aureus* in cord and adult blood samples after a 60-minute incubation, in the presence of either 3F6-hIgG1 or control hIgG1. It is noteworthy that *S. aureus* is the only known pathogen capable of coagulating plasma and agglutinating within fibrin cables, making accurate enumeration of bacteria in blood samples challenging [17]. To liberate bacteria from blood agglutinates, extracellular DNA traps, and host cells, blood samples were treated with streptokinase, nucleases and saponin, respectively prior to agar plating. Staphylococcal replication after 60-min incubation in blood was reported as the average of at least two measurements and calculated as the % of the initial bacterial inoculum by counting colony-forming units (CFUs).

**Fig. 2:**
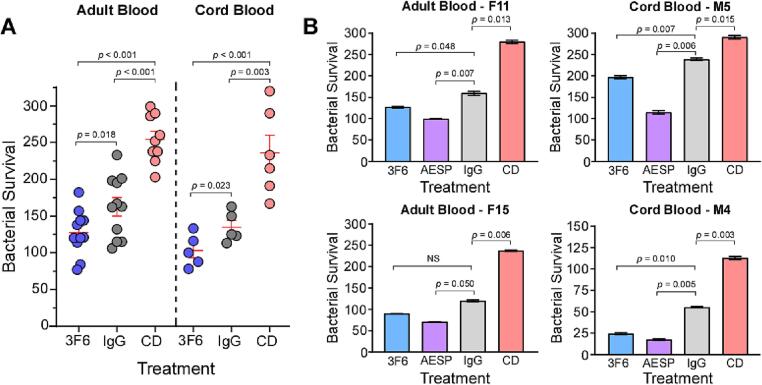
**Antibody enhanced phagocytic clearance of *S. aureus* in cord and adult blood.** (A) Bacterial survival in cord or adult blood pre-treated with 10 µg ml^−1^ 3F6-hIgG1 (3F6), 10 µg ml^−1^ control hIgG1 (IgG) or 0.04 mM Cytochalasin D (CD) after 60 min. The data represent the average ± SEM of colony forming units (CFU) after 60 min of incubation in blood, relative to the CFU of the initial inoculum (set as 100 %). Each condition was performed in duplicate and statistical significance was determined using unpaired independent Student’s *t* test. The data displayed represents experiments from 11 adult blood samples and 5 cord blood samples, conducted independently. (B) Bacterial survival in individual cord or adult blood samples after pre-treatment with 5 µg ml^−1^ 3F6-hIgG1 (3F6), 5 µg ml^−1^ 3F6-hIgG1^AESP^ (AESP), 5 µg ml^−1^ control hIgG1 (IgG) or 0.04 mM CD after 60 min. Incubations with each blood sample were performed in duplicate and statistical significance was determined using Dunnett's multiple comparisons test. Data displayed is representative of experiments from 5 adult blood samples and 3 cord blood samples, conducted independently.

Consistent with the notion that CD treatment blocks phagocytic uptake (Fig. 1B), a significant increase in CFUs was observed in cord blood treated with CD compared to control hIgG1 (134.8 % ± 9.3 % with hIgG1 vs 236.0 % ± 24.0 % with CD) (Fig. 2A). Adult blood samples treated with CD showed a similar trend, with higher CFU of staphylococci recovered after incubation compared to control hIgG1 (162.5 % ± 12.6 % with IgG1 vs 254.6 % ± 10.8 % with CD) (Fig. 2A). Bacterial counts were reduced by 24 % upon addition of 10 µg ml^−1^ of 3F6-hIgG1 to cord blood (103.0 % ± 9.9 % with 3F6-IgG1 vs 134.8 % ± 9.3 % with hIgG1) (Fig. 2). Similarly, a 22 % reduction was observed in adult blood with 10 µg ml^−1^ of 3F6-hIgG1 (127.4 % ± 9.2 % with 3F6-IgG1 vs 162.5 % ± 12.6 % with IgG1) (Fig. 2A). These findings suggest that cord blood effectively inhibits *S. aureus* growth through phagocytosis, comparable to adult blood. Furthermore, the addition of 3F6-IgG1 enhances this phagocytic uptake mechanism.

SpA molecules displayed on the surface of *S. aureus* bind to the Fcγ domains of IgG, including 3F6-hIgG1, which competes with Fcγ-Complement 1q (C1q) interaction, thereby diminishing complement activation and optimal opsonization of *S. aureus* [18]. Previously, we engineered 3F6-hIgG1 with four amino acid substitutions (AESP: S^254^A, Q^311^E, L^432^S, and N^434^P) abolishing the SpA-Fcγ domain interaction, thereby enhancing C1q, C3 and C4 recruitment on the surface of *S. aureus* [11]. Addition of 5 μg ml^−1^ 3F6-hIgG1^AESP^ further enhanced staphylococcal uptake compared to unmodified 3F6-hIgG1 in both cord blood and adult blood (Fig. 2B). Although cord blood exhibited slightly reduced levels of complement proteins C3/C4 compared to adult blood (Table 1), neonatal complement and immune cells retained the ability to effectively phagocytose and inhibit *S. aureus* growth in cord blood, similar to adult blood, and this capability was further enhanced with 3F6-hIgG1^AESP^.

**Fig. 3:**
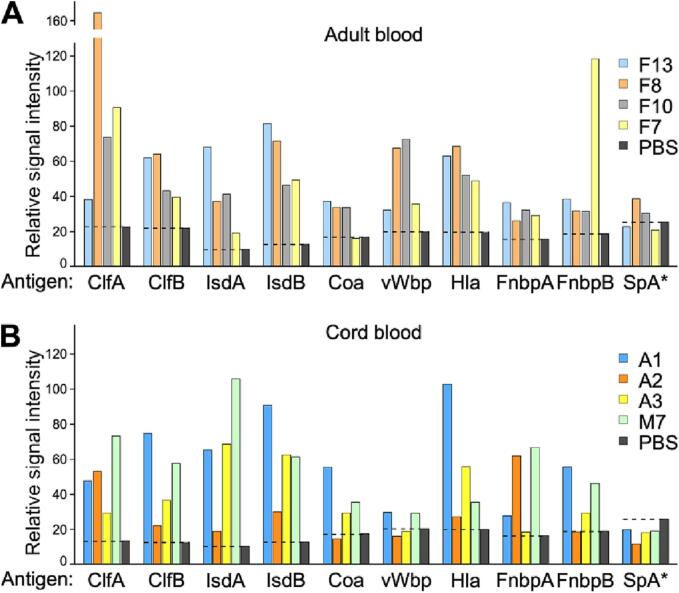
**Anti-*S. aureus* antibodies for representative donors.** Plasma samples collected from (A) adult blood and (B) umbilical cord blood were analyzed for antibodies against ClfA, ClfB, IsdA, IsdB, Coa, vWbp, Hla, FnbpA, FnbpB, and SpA_KKAA_. The dashed line represents the background signal obtained for PBS (in grey).

Using Enzyme-Linked Immunosorbent Assay (ELISA), we detected comparable levels of pre-existing *S. aureus* antibodies in both cord and adult blood plasma, suggesting successful maternal antibody transmission to cord blood donors (Fig. 3). However, the abundance of *S. aureus*-specific antibodies varied among donors and did not correlate with the extent of staphylococcal uptake in either cord blood or adult blood (Fig. 2). As anticipated, we measured negligible levels of antibodies to SpA (detected with SpA_KKAA,_ a variant of SpA that does not bind to Fcγ and Fab) in all of the donors.

## 4 Discussion

Susceptibility of newborns to invasive bacterial infections has been attributed to their immature immune system, impaired function of phagocytes, and hyperactive innate immune responses at birth [19]. Newborns exhibit lower levels of Ig and are heavily reliant on maternal IgG passively transferred across the placenta during the final trimester of pregnancy [20]. Neonatal T cells are as potent as adult T cells, but complement activity is reduced in newborns as compared to adults.

The failure of antibodies to protect against staphylococcal sepsis in clinical trials might be due to the inability of neonatal immune cells to eliminate the pathogen. Our study found a stronger association of *S. aureus* with neutrophils in anticoagulated cord blood than in adult blood. However, this association did not correspond to improved pathogen clearance. The increased association of *S. aureus* with neonatal neutrophils, without an accompanying improvement in bacterial clearance, may reflect an impairment in bactericidal activities. Yet, as with adult blood, the presence of 3F6-hIgG1 enhanced the uptake of *S. aureus* in cord blood; such uptake was further improved with 3F6-hIgG1^AESP^ even with an underdeveloped neonatal complement system, characterized by reduced levels of C1q, C4, C3, properdin, and factor B in newborns ([19] and Table 1).

While we detected substantial levels of pre-existing antibodies against *S. aureus* antigens, we hypothesize that these antibodies do not contribute to opsonophagocytic uptake as surface-bound SpA likely diverts them from their physiological targets. Consequently, a strategy neutralizing SpA, may be additive to pre-existing opsonizing antibodies and ultimately stimulate and enhance the neonatal immune system to combat staphylococcal infections.

## Declaration of Competing Interest

The authors declare that they have no known competing financial interests or personal relationships that could have appeared to influence the work reported in this paper.

## Data Availability

Data will be made available on request.
